# Effect of HT042, Herbal Formula, on Longitudinal Bone Growth in Spontaneous Dwarf Rats

**DOI:** 10.3390/molecules181113271

**Published:** 2013-10-28

**Authors:** Ji Young Kim, MiKyung Song, Donghun Lee, Jungbin Song, Sang Woug Park, Juyeon Park, Seungjoon Park, Ho-Young Choi, Hocheol Kim

**Affiliations:** 1 Department of Herbal Pharmacology, College of Korean Medicine, Kyung Hee University, Seoul 130-701, Korea; 2 Korea Institute of Science and Technology for Eastern Medicine (KISTEM), NeuMed Inc., Seoul 130-701, Korea; 3 Department of Pharmacology, School of Medicine, Kyung Hee University, Seoul 130-701, Korea

**Keywords:** HT042, herbal extract, longitudinal bone growth, spontaneous dwarf rat

## Abstract

HT042 is a new herbal prescription consisting of *Astragalus membranaceus*, *Phlomis umbrosa* and *Eleutherococcus senticosus*, which are used in Korean medicine to stimulate growth in children. We investigated the effects of HT042 on the body weight, longitudinal bone growth, and bone length in spontaneous dwarf rats (SDR). Male and female SDRs were divided into three groups: the control group (DW, 10 mL/kg/day), the recombinant human GH group (*rh*GH; 500 µg/kg/day), and the HT042 (100 mg/kg/day) group. Each group received the respective treatments for 10 days. Body weight was measured on day 10 of treatment. On day 8, tetracycline (20 mg/kg) was injected intraperitoneally into all individuals to form a fluorescent band on the newly synthesized bone. On day 10, femur and tibia lengths were measured using PIXImus. Body weight, longitudinal bone growth, and bone length were not affected in the HT042 group. In contrast, the *rh*GH group showed significantly increased body weight, longitudinal bone growth, and bone length. In conclusion, HT042 does not act through a GH-like effect to promote longitudinal bone growth.

## 1. Introduction

Growth retardation in children can be caused by endocrine, metabolic, nutritional, and/or genetic factors. Growth hormone (GH), metabolic acidosis, and malnutrition deficiencies are conditions that can interfere with a child’s ability to reach normal stature [1].

GH and insulin like growth factor-1 (IGF-1) are important regulators of longitudinal growth. The synthesis and release of GH from the anterior pituitary gland are promoted by growth hormone-releasing hormone (GHRH) and inhibited by somatostatin; however, GH is also regulated by a range of central and peripheral signals. IGF-1, which is secreted by the liver under GH control, inhibits GH secretion directly in somatotrophs and indirectly by stimulating the release of somatostatin [2]. GH circulates until it binds to a GH-binding protein, which is the extracellular domain of the GH receptor (GHR) [3]. The function of the GH-binding protein is not completely understood, although it may modulate the activity of GH either by prolonging its half-life or by reducing its availability to GHR.

The current methods for increasing final body height include GH treatment alone or combination with gonadotropin-releasing hormone (GnRH) agonists [4]. However, excessive GH treatment may increase the rate of growth, causing rapid progression through puberty, with the combination of GH and GnRH agonists often causing a decrease in bone mineral density (BMD) [5]. In addition, GH administration prevents the degradation of proteins that can occur in many diseases. Long-term treatment with GH increases lean body weight, muscle weight, and lipid weight while short-term treatment with GH increases plasma insulin and IGF-1 concentrations, and decreases protein concentrations in urine. However, the administration of GH is limited by its poor absorption from the gastrointestinal tract. Regarding the economics of GH treatment, biosynthetic GH is expensive, while the short- and long-term benefits for the individual are uncertain [6].

GH is also used in the management of other growth problems, such as idiopathic short stature and Turner syndrome. Overall, therapy is advantageous; however, there is also considerable variability in the response of patients with these diseases to GH, with some children remaining short even after treatment [7]. Thus, these studies demonstrate that although GH administration is beneficial for some children, it is not therapeutically effective in all patients.

Many ongoing studies are attempting to evaluate alternative therapeutic medicines [8]. Korean medicinal herbs have been used to treat children with growth retardation [9], and the results have been recorded in the traditional medicinal book *Dongeuibogam*. For the present bone growth study, we selected three herbs, the root of *Astragalus membranaceus*, the stem of *Eleutherococcus senticosus*, and the root of *Phlomis umbrosa*, and designated the resulting formula as HT042 [10].

HT042 was reported to increase the longitudinal bone growth, growth plate height, femur length, and nose-anus (N-A) and nose-tail (N-T) lengths in adolescent Sprague-Dawley (SD) rats. HT042 stimulated chondrocyte proliferation and chondrocyte hypertrophy in the growth plate, and directly increased the longitudinal tibia length and the N-A and N-T length in adolescent female rats. The heights of the growth plate and the chondrocyte zone provide direct evidence of longitudinal bone growth. HT042 stimulates cell proliferation and promotes chondrocyte transformation into a bone matrix, where longitudinal bone growth occurs [11].

In a previous study, HT042 was shown to increase longitudinal bone growth in SD rats to similar degrees as those observed with recombinant human GH (*rh*GH). In addition, it was confirmed that HT042 led to an increase in cell proliferation on the epiphyseal plate. The expression of BMP-2 and IGF-1 caused the length of the epiphyseal plate to grow. Furthermore, increased IGF-1 blood levels caused the femur length and body length to increase.

However, to our knowledge, there have been no studies on whether HT042 promotes longitudinal bone growth through a GH-like effect, or whether it functions through GHs to induce bone growth.

The spontaneous dwarf rat (SDR) is a dwarf strain derived from SD rats. SDR was reported to be a homozygous GH-deficient rat strain that is unable to produce normal GH mRNA because of a splicing abnormality [12]. Except for GH deficiency, SDRs have normal pituitary gland function, and thus serve as an appropriate model for studying GH-related effects [13]. In this study, therefore, we used the SDR model to investigate whether HT042 promotes longitudinal bone growth through a GH-like effect, or whether it is GH-dependent in its mechanism of achieving longitudinal bone growth.

## 2. Results and Discussion

### 2.1. Result

#### 2.1.1. Effect of HT042 on Body Weight in SDR

**Figure 1:**
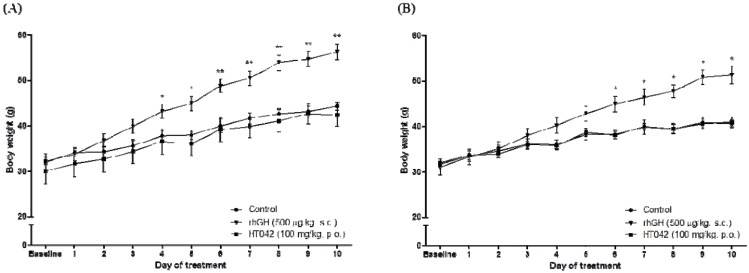
Effects of HT042 on body weight in spontaneous dwarf rats. (**A**) male, (**B**) female. The body weight of the animals was recorded daily during the experimental period. Data are means ± SD. * *p* < 0.05 or ** *p* < 0.01 compared with the control, by the Student’s *t* test.

**Figure 2:**
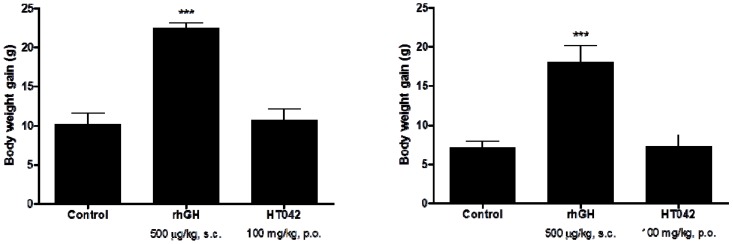
Effects of HT042 on body weight gain in spontaneous dwarf rats. (**A**) male, (**B**) female. The body weight of the animals was recorded daily during the experimental period. Data are means ± SD. * *p* < 0.05 or ** *p* < 0.01 compared with the control, by the Student’s *t* test.

Body weight increased over time in all groups. After the 10-day treatment period, the final mean body weight of the HT042-treated male and female groups were similar to control group (male, 44.3 ± 1.4 g *vs*. 42.4 ± 5.4 g; female, 40.6 ± 1.9 g *vs*. 41.0 ± 2.3 g). In comparison, a significant difference in body weight was observed in the *rh*GH-treated male and female groups at 4 and 6 days after initiating the experiment, respectively (male, 44.3 ± 1.4 g *vs*. 56.3 ± 3.7 g, *p* < 0.01; female, 40.6 ± 1.9 g *vs*. 51.3 ± 4.7 g, *p* < 0.05) (Figure 1 and Table 1). The body weight gain of the HT042-treated male and female groups was similar to the control group (male, 10.2 ± 1.4 g *vs.* 10.7 ± 1.4 g; female 7.1 ± 0.9 g *vs*. 7.3 ± 1.5 g). In comparison, the body weight gain of the *rh*GH-treated male and female groups was significantly greater compared to the control group (120.6% and 153.5%, respectively) (Figure 2 and Table 2).

**Table 1:** Effects of HT042 on the body weight in spontaneous dwarf rats.

Body weight (g)	Control	*rh*GH (500 µg/kg, s.c.)	HT042 (100 mg/kg, p.o.)
Male	44.3 ± 1.4	56.3 ± 3.7 **	42.4 ± 5.4
Female	40.6 ± 1.9	51.3 ± 4.7 *	41.0 ± 2.3

The body weight presented here is the final body weight. Data are mean ± SD values. * *p* < 0.05 or ** *p* < 0.01 compared with the control, by the Student’s *t* test.

**Table 2:** Effects of HT042 on body weight gain in spontaneous dwarf rats.

Body weight gain (g)	Control	*rh*GH (500 µg/kg, s.c.)	HT042 (100 mg/kg, p.o.)
Male	10.2 ± 1.4	22.5 ± 0.6 **	10.7 ± 1.4
Female	7.1 ± 0.9	18.0 ± 2.2 **	7.3 ± 1.5

The body weight gain was calculated by the equation: final body weight – initial body weight. Data are mean ± SD values. * *p* < 0.05 or ** *p* < 0.01 compared with the control, by the Student’s *t* test.

#### 2.1.2. Effect of HT042 on Longitudinal Bone Growth in SDR

**Figure 3:**
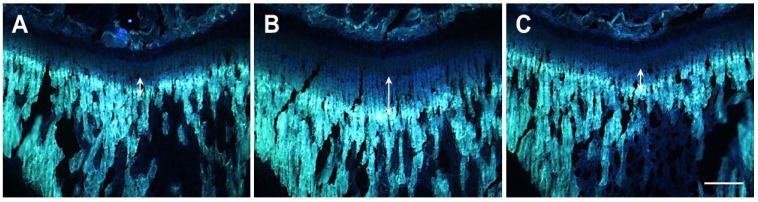
Fluorescence photomicrograph of the longitudinal section of the proximal tibia in spontaneous dwarf rats. The fluorescent line corresponds to the injection of tetracycline hydrochloride (20 mg/kg, i.p.). The arrow shows the amount of longitudinal growth. **A**: control group; **B**: recombinant human growth hormone (*rh*GH 500 µg/kg, s.c.)-treated group; **C**: HT042 (100 mg/kg, p.o.)-treated group. Scale bar = 200 µm

**Figure 4:**
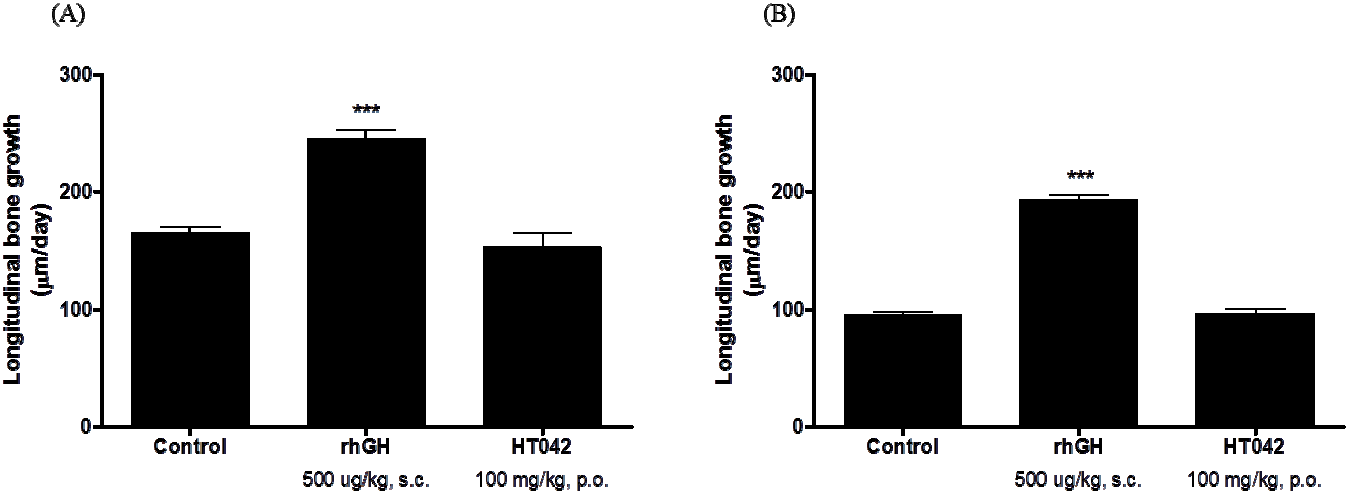
Effect of HT042 on longitudinal bone growth in spontaneous dwarf rats. (**A**) male, (**B**) female. Data are means ± SD. * *p* < 0.05, ** *p* < 0.01, or *** *p* < 0.001 compared to the control, by the Student’s *t* test.

The tetracycline injection administered to the rats in the present study formed a fluorescent line (Figure 3). The effect of HT042 treatment on longitudinal bone growth was assessed by measuring the gap between the successive band formed by tetracycline and the growth plate at three different locations, from which the average was obtained. The longitudinal bone growth in the control group of male SDRs was 164.4 ± 14.6 µm/day. The *rh*GH (500 µg/kg, s.c.)-treated group showed a significantly promoted longitudinal bone growth rate (245.0 ± 29.6 µm/day) compared to the control group (*p* < 0.001). The HT042 (100 mg/kg, p.o.)-treated group did not show a significantly promoted longitudinal bone growth rate (152.5 ± 39.4 µm/day) compared to the control group (Figure 4).

The longitudinal bone growth rate in the control group of female SDRs was 95.2 ± 8.2 µm/day. Females in the *rh*GH (500 µg/kg, s.c.)-treated group showed a significantly promoted longitudinal bone growth rate (193.0 ± 17.2 µm/day) compared to the control group (*p* < 0.001). Females in the HT042 (100 mg/kg, p.o.)-treated group did not show a significantly promoted longitudinal bone growth rate (96.7 ± 16.1 µm/day) compared to the control group (Figure 4).

#### 2.1.3. Effect of HT042 on SDR Bone Length

**Figure 5:**
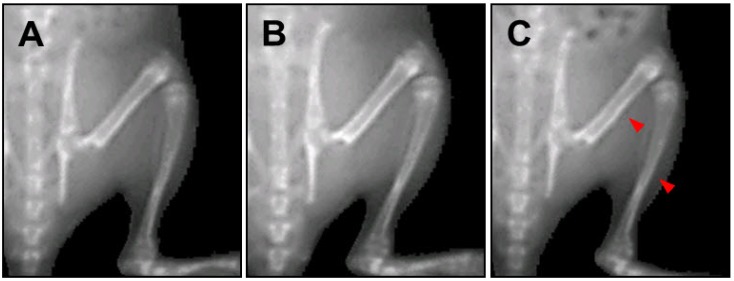
Images of bone by PIXImus on spontaneous dwarf rats. The arrows indicate the area where bone length was measured in the femur and tibia. **A**: control group; **B**: recombinant human growth hormone (*rh*GH, 500 µg/kg, s.c.)-treated group; **C**: HT042 (100 mg/kg, p.o.)-treated group.

We used PIXImus to measure the femur and tibia length before sacrificing the rats, to serve as a skeletal growth marker (Figure 5). The femur length of the HT042-treated male and female groups was similar to control group. In comparison, a significant difference in femur length was observed in the *rh*GH-treated male and female groups (10.2% and 6.4%, respectively) compared to the control group. The tibia length of the HT042-treated male and female groups were similar to control group. In comparison, a significant difference in tibia length was observed the *rh*GH-treated male and female groups (7.5% and 5.7%, respectively) compared to the control group (Table 3).

**Table 3:** Effects of HT042 on the bone length of spontaneous dwarf rats.

Length (mm)	Control	*rh*GH (500 µg/kg, s.c.)	HT042 (100 mg/kg, p.o.)
Femur			
Male	16.6 ± 0.7	18.3 ± 0.9 *	16.4 ± 1.2
Female	18.8 ± 0.3	20.0 ± 0.2 **	18.9 ± 0.3
Tibia			
Male	24.0 ± 0.7	25.8 ± 0.9 *	23.7 ± 1.3
Female	23.0 ± 0.5	24.3 ± 0.5 **	22.6 ± 0.7

The bone lengths presented here are the final bone lengths. Data are mean ± SD values. * *p* < 0.05 or ** *p* < 0.01 compared with the control, by the Student’s t test.

### 2.2. Discussion

In the present study, it was found that HT042 does not affect the body weight, the longitudinal growth of long bones (femur and tibia), or the longitudinal bone growth in SDRs.

In a previous study, the body weight of SDRs was inhibited by the absence of GH in the anterior pituitary [14]. In this study, HT042 did not influence SDR body weight gain. The body weight of the rhGH-treated group significantly increased by 27.1% (male) and 26.4% (female) compared to the control group. Other studies on SDRs have reported an increase in body weight following GH treatment [15]. These findings indicate that HT042 does not have a GH-like effect.

In this study, the longitudinal bone growth of the tibia was assessed by measuring the length between the fluorescent line formed by tetracycline and the epiphyseal end of the growth plate. The longitudinal growth of the tibia was similar in the HT042-treated group and the control group. Hence, HT042 did not affect longitudinal bone growth in SDRs. The longitudinal growth of the tibia in the *rh*GH-treated group was significantly greater by 6.4% (males) and 7.4% (females) compared to the control group. These results support that HT042 does not have a GH-like effect.

Several hormones are important for normal postnatal longitudinal bone growth; however, it is generally accepted that GH is the most important hormone in this respect. Furthermore, it has been demonstrated that GH stimulates the growth of cartilage and other tissues by increasing the number of cells, rather than by increasing cell size [13,16,17,18,19]. A widely discussed question over the last two decades has been whether GH acts directly on tissues, or whether the effect of GH is mediated by a liver-derived growth factor. This growth factor was initially called sulfation factor, but was later renamed somatomedin and subsequently shown to be identical to IGF-I. According to the original somatomedin hypothesis, GH promotes skeletal growth by stimulating the production of somatomedin in the liver, which, in turn, promotes longitudinal bone growth in an endocrine-like manner [13,20,21,22].

In this study, the lengths of the femur and tibia were not affected by HT042 administration to SDRs. The lengths of both the femur and tibia significantly increased in SDRs treated with *rh*GH compared to the control group. The femur length of the *rh*GH-treated group significantly increased by 10.2% (male) and 6.4% (female) compared to the control group. The tibia length of the *rh*GH-treated group significantly increased by 7.5% (male) and 5.7% (female) compared to the control group. Other studies on the SDR have reported an increase in bone length following GH treatment [15]. These results indicate that HT042 did not have a GH-like effect.

SDRs have normal pituitary function, except for GH deficiency, and, hence, are probably the most appropriate models for studying the specific effect of GH deficiency [13]. SDRs originated from a normal SD rat strain, and have been widely used in experimental studies involving laboratory rats. The SDRs are a mutant strain that lack GH in both the anterior pituitary gland and blood plasma [23]. The absence of pituitary and circulating GH is the result of an autosomal recessive mutation in the GH gene in the somatotrope cells of the anterior pituitary gland, which produces an abnormal splice variant, resulting in premature translational termination. As a result, GH protein is not synthesized, and its absence in both the somatotrope cells and blood plasma [23,24].

In a previous report, the HT042 herbal formula exhibited synergistic effects in promoting longitudinal bone growth. Consistent with the previous study, *E. senticosus* and *A. membranaceus* increased bone growth; however, each of the component herbs of HT042 showed lower efficacy compared with the HT042 [25]. *E. senticosus* was reported to induce the longitudinal bone growth in adolescent rats [26] and to stimulate BMD of the mouse tibia [27]. *A. membranaceus* was reported to stimulate the proliferation and promotion of bone marrow stromal cells [28] and to promote GH in pituitary cell culture [29]. We demonstrated that a 4-week treatment with HT042 significantly promoted axial skeletal growth in adolescent female rats. This long-term treatment effect on skeletal growth was consistent with the short-term treatment effect. HT042 has been reported to increase the BrdU cell proliferation, bone morphogenetic protein-2 (BMP-2), and insulin-like growth factor-1 (IGF-1) expression on the growth plate, and IGF-1 concentration in serum. HT042 increases the overall growth plate height responsible for transformation into bone matrix, where longitudinal bone growth occurs [11]. HT042 stimulates cell proliferation of chondrocytes. The longitudinal bone growth effect of HT042 is induced by local IGF-1 and BMP [30,31]. HT042 and each herb used in HT042 do not affect uterine hypertrophy. HT042 has no uterotrophic effects on sexually immature and mature rats. HT042 does not induce the senescence of the growth plate through estrogenic activity. HT042 induces cell proliferation and bone growth at the epiphyseal plate without estrogenic activity [10,25]. Previous research has shown that HT042 promotes longitudinal bone growth in normal rats. However, it does not cause longitudinal bone growth in SDRs deficient in GH.

## 3. Experimental

### 3.1. Plant Material

The dried roots of *P. umbrosa*, the roots of *A. membranaceus* Bunge, and the stem barks of *E. senticosus* Harms were used (Omniherb Co., Daegu, Korea). These materials were identified by Professor Dr. Hocheol Kim, and the voucher specimens were deposited in the Department of Herbal Pharmacology, College of Oriental Medicine, Kyung Hee University, Seoul, Republic of Korea.

### 3.2. Preparation of Samples

The dried roots of *P. umbrosa*, the roots of *A. membranaceus* Bunge, and the stem barks of *E. senticosus* Harms were extracted separately with 70% ethanol for 6 h at 80 °C in a reflux apparatus. After refluxing, the extracts were filtered, the filtrates were evaporated in a rotary evaporator, and the samples were lyophilized in a freeze dryer (Operon, Gyeonggi, Republic of Korea). The extract yields of *P. umbrosa*, *A. membranaceus*, and *E. senticosus* were 22%, 14%, and 6%, respectively. Then, the resulting powders were mixed at a ratio of 26.5:31.2:42.3. The quantitative authentication of HT042 was performed on a Waters instrument (Waters, Milford, MA, USA) equipped with a Waters 1525 binary pump, a Waters 2707 autosampler, and a Waters 2998 PDA detector using a Sunfire C18 column (5 μm; 250 × 4.6 mm; Waters, USA). The gradient system involved 2 mobile phases, A and B. The gradient elution in mobile phase A contained 0.5% phosphoric acid. The gradient elution in mobile phase B contained acetonitrile. The former delivered at a flow rate of 1.0 mL/min for *P. umbrosa* and *E. senticosus*, at 0–20–30–40 min and 5%–17%–22%–30%. The latter delivered the same flow rate for *A. membranaceus* at 0–15–25–28–30 min and 35%–35%–65%–35%–35%. Each extract was analyzed in triplicate.

**Figure 6:**
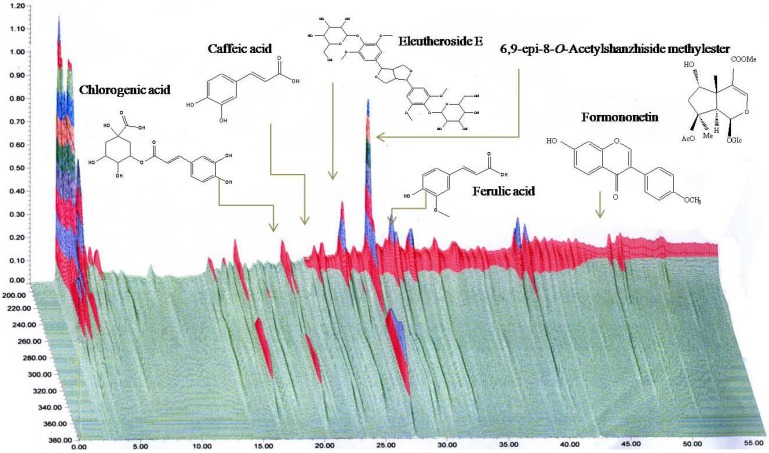
Three-dimensional high-performance liquid chromatogram of HT042, a blend of three herbal extracts.

The high-performance liquid chromatogram of HT042 is shown in Figure 1. The quality of HT042 was standardized using three representative components: 315.46 ± 33.58 mg% of 6,9-*epi*-8-*O*-acetylshanzhiside methyl ester for *P. umbrosa*, 14.32 ± 0.17 mg% of formononetin for *A. membranaceus*, and 298.73 ± 31.95 mg% of eleutheroside E for *E. senticosus*. The mixed extracts were used as sample materials and stored at −20 °C until use (Figure 6).

### 3.3. Animals

To investigate the effects of the HT042 treatment on longitudinal bone growth, 3-week-old SDRs were used. SDRs were obtained from the Department of Pharmacology, College of Medicine, Kyung Hee University (Seoul, Republic of Korea). The experimental procedures were performed in accordance with the animal care guidelines of the Kyung Hee University institutional animal care and use committee (protocol no. KHUASP (SE)-10-034). The animals were housed under controlled temperature (23 ± 2 °C), relative humidity (55 ± 10%) and lighting conditions (lights on from 07:00 to 19:00), with food and water made available *ad libitum*.

### 3.4. Treatments

SDRs (male n = 13 and female n = 15) were randomly allocated into three groups according to supplement regimen; namely, the control group, the HT042 group, and the *rh*GH group. The control treatment (Distilled water, DW; male n = 3 and female n = 4) and the HT042 treatment (100 mg/kg; male n = 5 and female n = 5) were administered twice daily at 09:00 and 21:00 in a fixed dose volume of 10 mL/kg. The *rh*GH treatment (500 µg/kg; male n = 5 and female n = 6) (LG, Seoul) was subcutaneously injected once daily. All three treatments were administered daily for 10 consecutive days in each case. On day 8, all animals were injected intraperitoneally with tetracycline hydrochloride (20 mg/kg, Sigma, St. Louis, MO, USA) in distilled water to measure longitudinal bone growth. Forty-eight hours after the injection, all animals were sacrificed, and then the tibias were dissected and all soft tissue was removed. Body weight was recorded at the same time every day over the 10-day treatment period.

### 3.5. Tissue Preparation and Detection of Longitudinal Bone Growth

The dissected tibias were fixed in 4% paraformaldehyde for 48 h, and dehydrated by immersion in 30% sucrose for 1 day. Dehydrated bone was directly sectioned longitudinally at a thickness of 40 µm with a microtome (Leica, Berlin, Germany). The sections were mounted onto gelatinized glass slides, and observed by fluorescence microscopy (Olympus, Tokyo, Japan), to measure the longitudinal bone length between the fluorescent line and the epiphyseal end line of the growth plate at 3 different locations using ImageJ software (version 1.45, National Institute of Health, Bethesda, MD, USA). Data were averaged to calculate longitudinal bone growth rates.

### 3.6. Bone length Measurements

Bone length measurement was obtained by the PIXImus (GE Lunar PIXImus, GE Healthcare, Madison, WI, USA) before the rats were sacrificed. The rats were anesthetized with N_2_O/O_2_ gas and isoflurane. The quantity of anesthetic was sufficient to keep the rat anesthetized for the period of the scan (approximately 5 min). After completion of the scan, the anesthetized rats were placed in a stretched position, so that their body lengths could be measured. The bone length between the femur and tibia was measured using ImageJ software (version 1.45, National Institute of Health, Bethesda, MD, USA). Data were averaged to calculate the bone length.

### 3.7. Statistical Analysis

All the data were presented as mean ± SD. The effects of the different treatments were compared by Student’s t test using GraphPad Prism 5 (GraphPad Software Inc., La Jolla, CA USA). *p* < 0.05 was considered statistically significant.

## 4. Conclusions

The results of the current study clearly show that HT042 does not act through a GH-like effect to promote longitudinal bone growth.
